# Numerical Simulation of Failure Behavior of Granular Debris Flows Based on Flume Model Tests

**DOI:** 10.1155/2013/603130

**Published:** 2013-05-14

**Authors:** Jian Zhou, Ye-xun Li, Min-cai Jia, Cui-na Li

**Affiliations:** ^1^Department of Geotechnical Engineering, Tongji University, Shanghai 200092, China; ^2^Key Laboratory of Geotechnical and Underground Engineering of Ministry of Education, Tongji University, Shanghai 200092, China

## Abstract

In this study, the failure behaviors of debris flows were studied by flume model tests with artificial rainfall and numerical simulations (PFC^3D^). Model tests revealed that grain sizes distribution had profound effects on failure mode, and the failure in slope of medium sand started with cracks at crest and took the form of retrogressive toe sliding failure. With the increase of fine particles in soil, the failure mode of the slopes changed to fluidized flow. The discrete element method PFC^3D^ can overcome the hypothesis of the traditional continuous medium mechanic and consider the simple characteristics of particle. Thus, a numerical simulations model considering liquid-solid coupled method has been developed to simulate the debris flow. Comparing the experimental results, the numerical simulation result indicated that the failure mode of the failure of medium sand slope was retrogressive toe sliding, and the failure of fine sand slope was fluidized sliding. The simulation result is consistent with the model test and theoretical analysis, and grain sizes distribution caused different failure behavior of granular debris flows. This research should be a guide to explore the theory of debris flow and to improve the prevention and reduction of debris flow.

## 1. Introduction 

Debris flows are rapid mass movements of water and debris. They are often triggered by heavy or prolonged rainfall in mountainous area with regolith surface. As debris flow has great potential energy to move, it would result in a huge hazard which causes significant damage and economic losses.

Due to translational or rotational failure of saturated or undercut slopes, debris flow often occurs with different grain sizes distributions in soil. Various experimental models were conducted to simulate the debris flow at the formation mechanism [1–3], movement and deposition [4], disaster prevention and mitigation [5], and dynamic constitutive model [6, 7] of rainfall debris flow. Wang and Sassa [8, 9] study the relationship between particle sizes and damage level of soil with different sand particles of debris flow. Dahal et al. [10] and Ochiai et al. [11, 12] have, respectively, studied the soil movement, bulk strain, and pore water pressure during the slope sliding. All these researches show that grain size distributions can have profound effects on the hydrologic response of watersheds by changing the infiltration characteristics and erodibility of the soil, which leads to decreased rainfall infiltration and increased overland flow and runoff in channels significantly. So, the grain size distribution is one point of this paper.

Through appropriate simplifications, assumptions, and computation schemes, most of the numerical models of debris flows can be mathematically formulated on the basis of mass and momentum conservation equations incorporating with material rheology [13–15]. The potential advantages of discrete element method (DEM) at debris flow was proved by Asmar et al. [16] through his simulation of three-dimensional particle flow to get the process of debris flow of flow stress distribution and energy changes. Valentino et al. [17] analyzed the flow of dry sand through the indoor small-model test and DEM. By using two-dimensional particle flow code program (PFC^2D^), Hu et al. [18] analyzed the formation process of debris flow with detrital material and the relationship between soil and moisture content under the action of rainfall. Based on the molecular dynamics, De Blasio [19] put forward a numerical simulation method, which is simple and convenient simulation viscous debris flow, through increasing the viscous force of the DEM particles. Data from those researches indicate that the discrete element method (DEM) can have profound effects on the mechanism of debris flow by overcoming the hypothesis of the traditional continuous medium mechanics and considering the simple characteristics of particle. So, the particle flow code (PFC^3D^) program was chosen in this paper to simulate the formation process of debris flow.

The failure behaviors of debris flow were studied with different grain size distributions by using laboratory flume model tests and numerical simulation (PFC^3D^). In the flume model tests, sand samples were prepared by fine or medium sand with different mixing ratio. Combining with the digital imaging technology, the failure behaviors were analyzed in deformation and displacement field of debris flow slope, and the failure mode of debris flow tests was carried with different grain size distributions. In numerical simulation (PFC^3D^), the numerical simulation model of debris flow was conducted based on the discrete element method of PFC^3D^. The numerical simulation model can better reflect the formation of debris flow when compared with the results of laboratory tests. At last, the failure behaviors mechanism of debris flow was studied by PFC^3D^. Based on flume model and numerical tests, the failure mode of debris flow with different grain size distributions was summarized. This research should be a guide to explore the theory of debris flow and to improve the prevention and reduction of debris flow.

## 2. The Flume Model of Debris Flow

The flume model tests were carried out by self-designed flume model device. During the experimental process, digital photos of the debris flow were taken at different stage in order to get information of failure behavior of debris flow slope.

### 2.1. Model of Debris Flows

**Figure 1:**
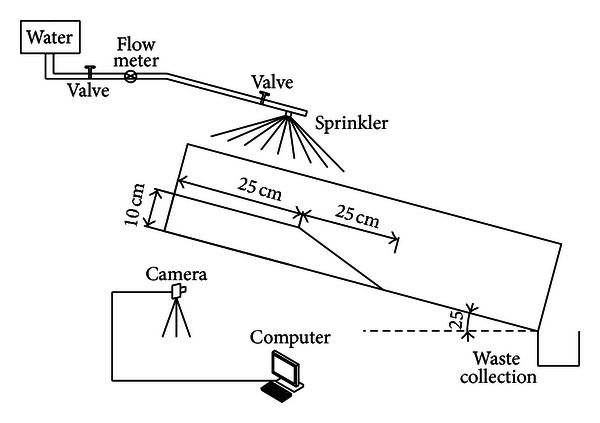
Debris flow test system.

The flume model of debris flow was shown in Figure 1. The test system is composed by three parts: (1) flume model, (2) artificial precipitation device, and (3) measurement and data logger. The digital pictures were taken during the failure process of slopes. The size of flume is 150 cm in length, 40 cm in width, and 25 cm in height.

### 2.2. Slope Preparation

**Figure 2:**
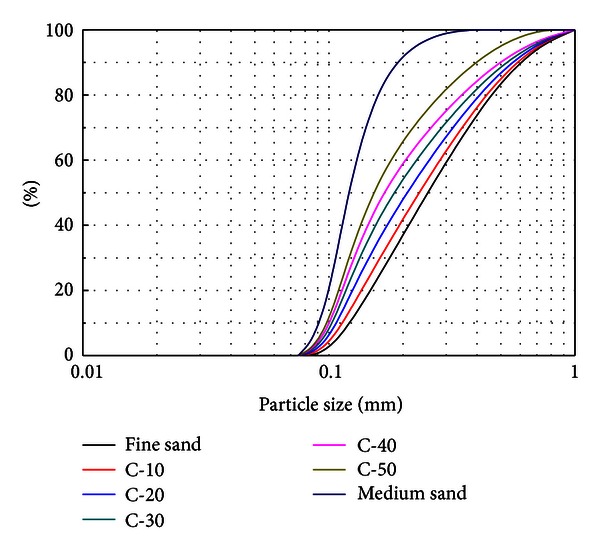
Grain size distributions.

Sand samples were prepared by mixing medium sand (*D*_50_ = 0.35 mm) and fine sand (*D*_50_ = 0.15 mm). The grain size distribution curves of medium and physical indices of fine sands are shown in Figure 2 and Table 1, respectively.

**Figure 3:**
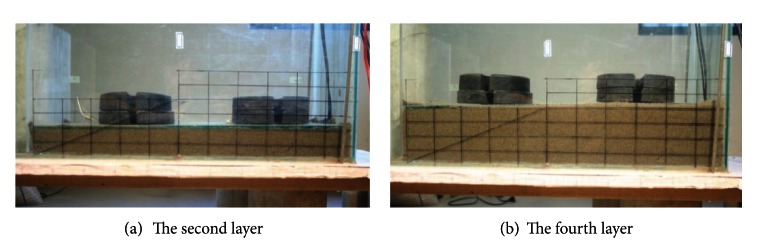
Weight applied to let the soil settle.

The flume was set horizontally as Figure 3 indicates. Sand samples were dried and mixed with water to reach an initial water content of 10%. A thin layer of medium sand was glued on the bottom of the flume to increase the friction between sand and the flume. Sand was laid into the flume with four layers using falling sand method with a thickness of 2.5 cm for each layer. Before the preparation of successive layer, a 20 kg mass was applied evenly on the sand surface for 1 hour to make the soil settle. And the test began after the last layer has completed for 18 hours. Then the flume was tilted to the designed angle, 25 degree for the test. The rainfall intensity for each test was kept at 1 mm/min. The duration of rainfall for the tests was within the range from 6.1 min. to 7.1 min.

## 3. Experimental Results Analysis

### 3.1. The Flume Tests

**Figure 4:**
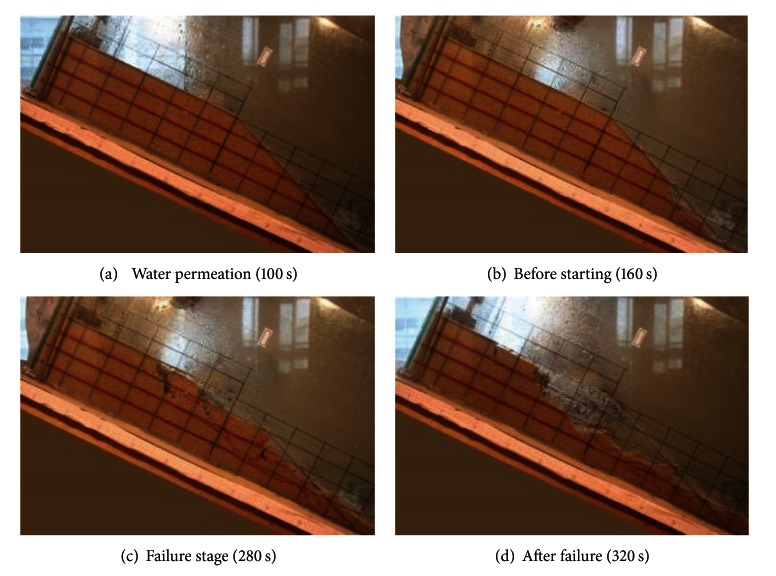
The failure process of debris flow (side view).

Based on the experimental observation, the failure process of debris flow can be divided into four stages as below. The failure process of medium sand slope under artificial rainfall is shown in Figure 4.

**Figure 5:**
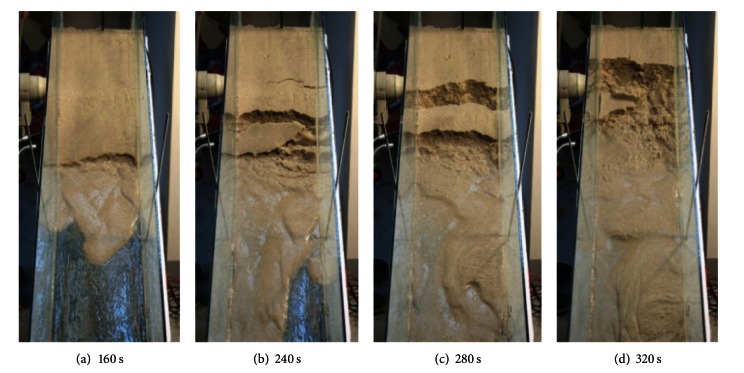
The failure process of debris flow (top view).

At the water infiltration stage as shown in Figure 4(a), there is no obvious surface settlement occurred at slope surface with the increases of soil moisture content. At the starting stage as shown in Figure 4(b), shearing deformation is observed at sandy slope toe soon after soil saturated. After the sliding of slope foot sand, new shearing deformation is found in the posterior slope. At the failure stage as shown in Figure 4(c), tension cracks appeared on the surface of slope and it developed into shear sliding surface with water flow. With the sliding down of shear surface, new tension crack and shear sliding surface are found again in the posterior slope. At the after failure stage as shown in Figure 4(d), the collapsed sand particle and water accelerated moving down under gravity and seepage force. Debris flow is formed with sliding sand and flowed water.  Figure 5 is the top view of the failure process of debris flow.

Through the above analysis, it is found that the failure in slopes starts with shear deformation at the slope toe and takes the form of retrogressive toe sliding failure.

### 3.2. Displacement Field

**Figure 6:**
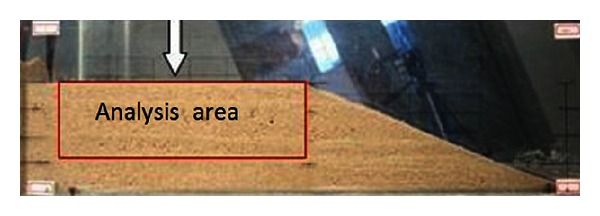
The analysis area of displacement field.

Full-field displacement can be acquired by digital methods [20]; in this paper it was analyzed by digital photogrammetry for deformation measurement (DPDM). The DPDM technique has been proven to be a powerful tool for observing the process and local deformation of granular soil [21]. Local area was chosen to analyze displacement of debris flows slope as indicated in Figure 6.

**Figure 7:**
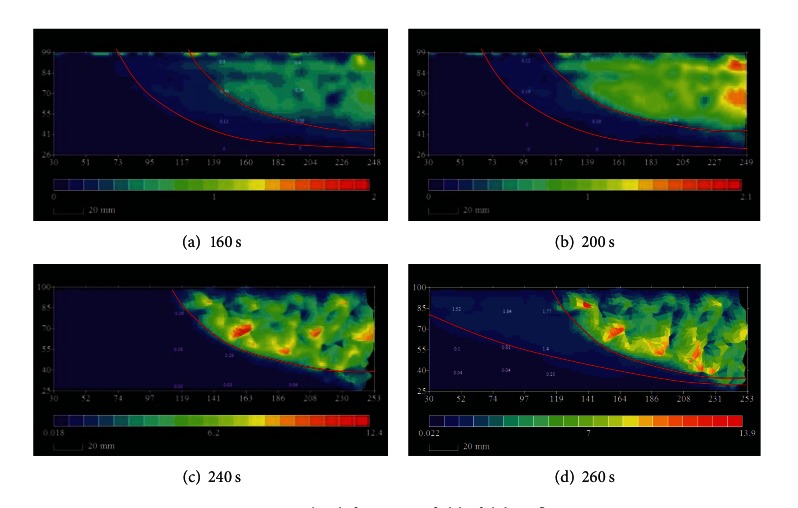
The deformation field of debris flow.

Figure 7 shows the displacement field in different colors of debris flows, and the colors representing displacements are shown under each figure. As Figure 7(a) indicates, two potential sliding surfaces are found in the 5.0~7.0 cm deep. Soil above potential sliding surfaces can be considered as sliding mass which has a downward trend. As rainfall continues, the shape of sliding mass keeps no change, but the displacement of the upper slope has greater increase, and the largest displacement in Figure 7(b) is 9.1 mm. However, the displacement of slope outside sliding surfaces still remains in 0 mm. With the working of seepage water and losing the support of sandy slope toe, the stability of slide mass is distorted as shown in the Figure 7(c). At this stage, sliding mass is divided into small parts and slide down with greater displacement. When the old slide mass disappeared, new sliding surfaces are found in Figure 7(d), which means that the failure cycle will be repeated.

The evolution process of displacement field analysis was confirmed with the flume model test, and the failure of granular debris flow is slopes collapsing in form of slide mass with greater displacement.

### 3.3. Failure Mode

In order to study the relationship of grain size distributions and failure mode of debris flow, seven sand samples were prepared in this paper by mixing medium and fine sand. Fine sand contents in a sample are 0% (medium sand), 10% (C-10), 20% (C-20), 30% (C-30), 40% (C-40), 50% (C-50), and 100% (fine sand), respectively. And the grain size distributions of different sand samples are shown in Figure 2.

**Figure 8:**
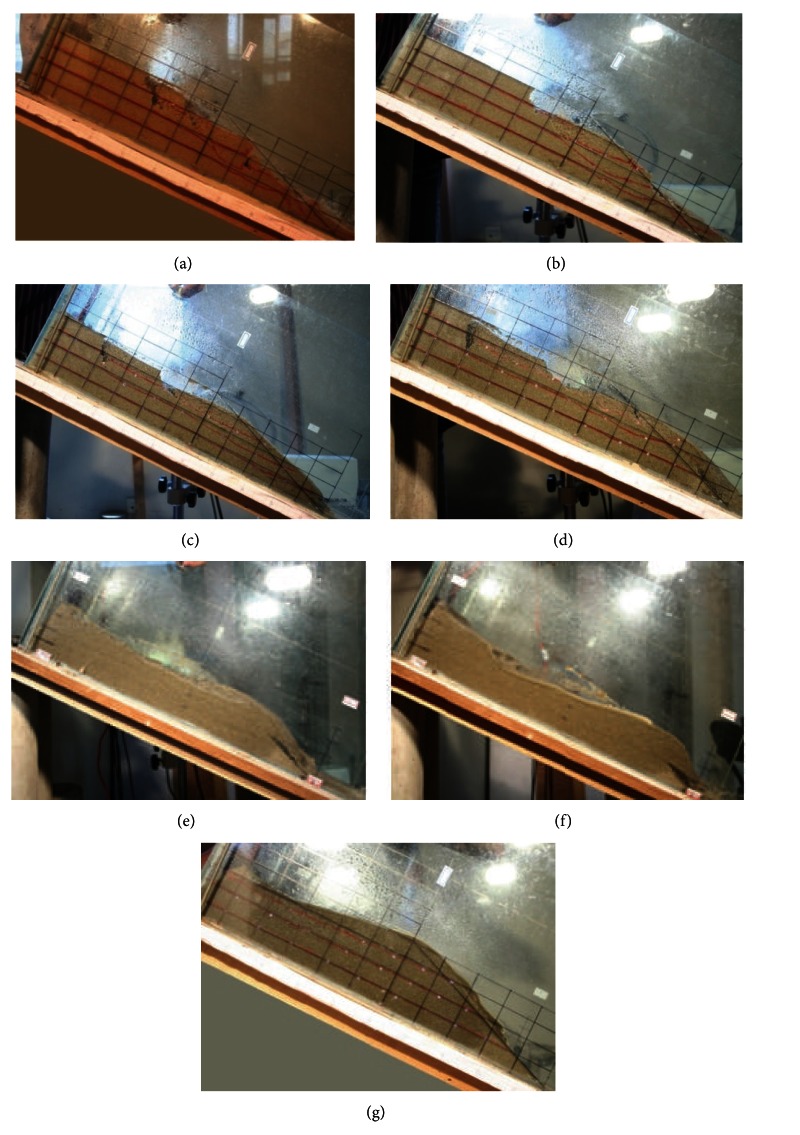
Failure of sand slopes with different fine particle contents: (a): medium sand, (b): 10% of fine sand, (c): 20% of fine sand, (d): 30% of fine sand, (e): 40% of fine sand, (f): 50% of fine sand, and (g): fine sand.

Pictures were taken during the tests to record the progress of the failure of each slope. The results are shown in Figure 8. The failure in slopes with minor fine particle contents as less 10% (Figures 8(a) and 8(b)) starts with cracks at crest and takes the form of retrogressive toe sliding failure. The slide surface is circular, and the sliding part is small and travels only a short distance. With the increase of fine particles in the soil, the failure mode of the slopes changed to fluidized flow when the fine particle content is more than 40% (Figures 8(e), 8(f) and 8(g)). With this type of failure, a large part of the slope slide down the slope like viscous fluid. When fine particle content is within the range of 20% and 30% (Figures 8(c) and 8(d)), the failure starts with cracks at the crest and flows down the slopes. The description of the failure modes is discussed in Section 4.4. The failure descriptions of sand slopes with different contents of fine sand were summarized in Table 2.

The flume model tests indicated that the content of fine sand in mixed slope influenced the failure behavior of granular debris flows as the slope has greater flowability with more fine sand content. When the content of fine sand increased from 10% to 100%, the failure mode of granular debris flows changed from retrogressive toe sliding failure to fluidized flow.

## 4. Numerical Simulation

The innovation of numerical mode used in this study was liquid-solid coupled method [22]. This scheme solves the continuity and Navier-Stokes equations for incompressible fluid flow numerically in an Eulerian Cartesian coordinate system and then derives the pressure and fluid velocity for each fixed grid (or cell) by including the influence of particles, and the corresponding porosity, within each cell. Driving forces from the fluid flow are applied to the particles as body forces. These forces are also added to the fluid equations and cause change in momentum, as reflected by the change in the pressure gradient in the flow direction.

### 4.1. Numerical Model

**Figure 9:**
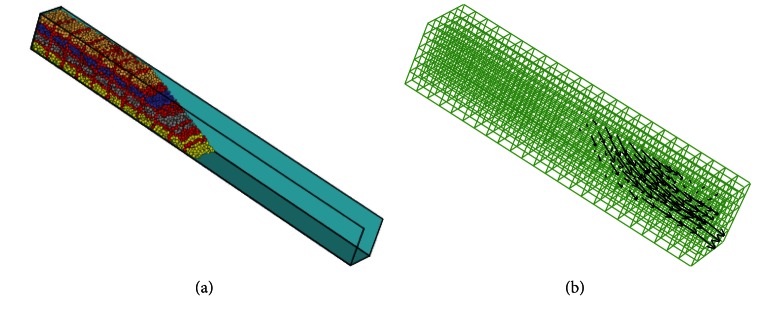
The numerical model (PFC^3D^) and fluid grid cell of debris flow numerical model.

Numerical model in Figure 9(a) is 1.0 m in length, 0.1 m in width, and 0.1 m in height. The particle sizes of numerical sample are 2.0 to 5.0 mm with a median diameter (*D*_50_ = 3.5 mm) which is 10 times bigger than model experimental sand samples (*D*_50_ = 0.35 mm).

In numerical model, fixed coarse-grid fluid model (Figure 9(b)) was used to simulate rainfall, and the impact of flow water on particle was realized by changing friction coefficient and damping coefficient. As the experimental sand sample is nonsaturated soil, contact adhesive model was used to simulate matric suction of nonsaturated soil cohesion. Contact stiffness and slip model were considered in the numerical model of debris flow. The coefficient used in flume numerical model is shown in Table 3.

### 4.2. Comparison Authentication

**Figure 10:**
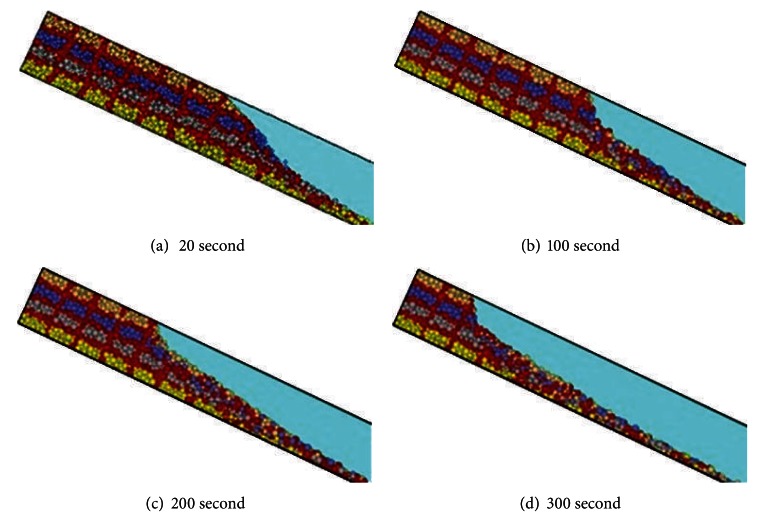
The formation process of debris flows of numerical simulation.

Based on the numerical model (PFC^3D^), the failure process of slope was simulated. In order to facilitate the observation, the slope was partitioned by red particles in the numerical model, and the slope was divided into four layers by red, grey, blue, and yellow color particles.  Figure 10 shows the failure phenomenon during the different stages of failure process of debris flow of numerical simulation. In the numerical simulation, the slide of slope begins at the slope toe, and particles slide down from slope toe to the back of slope in layer-by-layer. The numerical simulation indicated that the failure of debris flow starts with slide at slope toe and takes the form of retrogressive toe sliding failure. Comparing the result of numerical simulation and the flume model tests of debris flow (Figure 4), the formation process for debris flow of numerical simulation is similar to the flume model tests. It demonstrated that it is satisfactorily to simulate the three-dimensional behavior of flume model results of granular debris flow.

The results of numerical simulation indicated that the established numerical model could reflect the formation process of granular debris flow, and this numerical model can do in-depth study on failure behavior of granular debris flows.

### 4.3. Displacement Analysis

**Figure 11:**
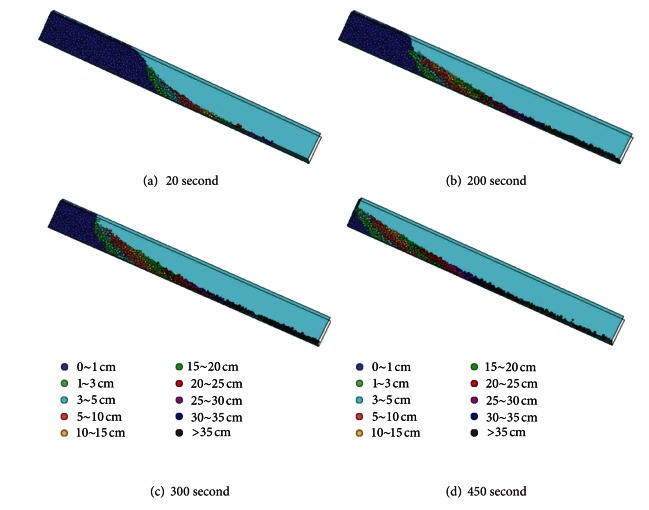
The deformation field of debris flow.

The displacement output by the numerical model (PFC^3D^) is divided into ten colors which represent different displacement, and the color legend was showed in the right of Figure 11. Figure 11 shows the particles displacement during the different stages of failure process of debris flow. Under the seepage force and gravity, shearing deformation appears at slope toe at first. When particles at the slope toe reached seepage failure, particles slide down in layers at the work of seepage force and lose the support of slope foot. The particle at failure area slide down quickly with greater displacement. During the failure particles sliding down, particles at the upper layer have bigger displacement while particles at the bottom layer have smaller displacement. The failure process of numerical simulation is tiered slide which fits with the flume model tests.

The displacement of slope which clearly shows the failure process of granular debris flow indicates that the slope has tiered slide from front to back of slope and takes the form of retrogressive toe sliding failure, and particles at the upper layer have greater displacement than particles at the bottom layer.

### 4.4. Failure Mode Comparison

**Figure 12:**
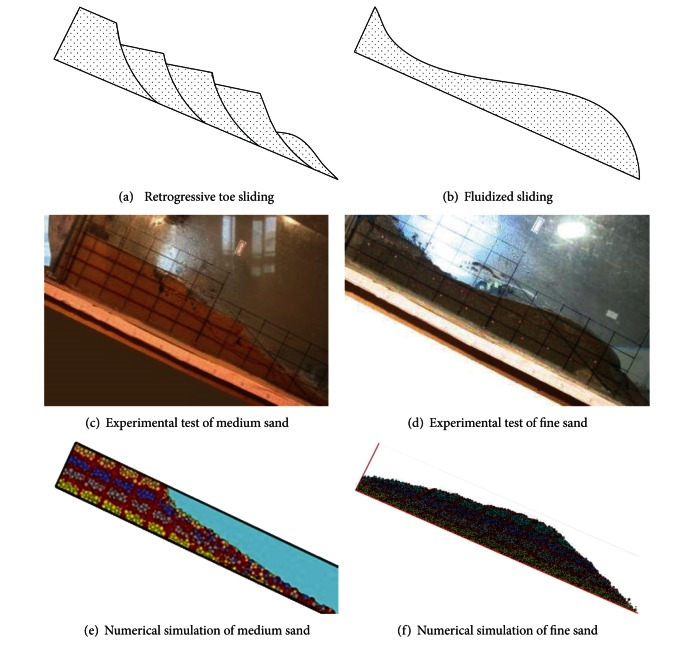
The failure mode of debris flow slope with medium sand or fine sand.

Based on the experimental test and numerical simulation, grain size distributions (fine sand) cause different failure behavior of granular debris flows, and the failure modes of medium and fine sand slopes are, respectively, retrogressive toe sliding and fluidized sliding represented in Figure 12. The figures show clearly the difference between the retrogressive toe sliding (Figure 12(a)) and fluidized slide (Figure 12(b)). The retrogressive failure of medium sand slope is tiered sliding. The slope slide begins at the foot of slope, and the upper particles slide down as losing the support of lower part. The retrogressive failure confirms the results of experimental test [9]. The fluidized slide of fine sand slope is whole body sliding in a short time as viscous fluids. The fluidized slide is in good agreement with the results of experimental test and theoretical derivation [9, 23].

The analysis of failure behavior of granular debris flows indicated that grain size distributions (fine sand) have an important influence on failure behavior of granular debris flows. Fine sand fills in the blanks among the large sand particle, which lead to high pore water pressure within the soil and make the soil slope have greater flowability [24]. Grain size distributions can have profound effects on the hydrologic response of watersheds by changing the infiltration characteristics and erodibility of soil, which leads to decreasing rainfall infiltration. Slope with different fine content has different permeability coefficient which result in different seepage velocity of water in soil. Therefore, the permeability characteristics of soil with different grain size distributions and the pore water pressure change during the failure process of granular debris flow should be studied to discover the relationship between the permeability, pore water pressure, and failure behavior of granular debris flows in the subsequent research work.

## 5. Conclusion

Experimental tests identify that grain size distributions can have profound effects on failure mode of granular debris flow, and the failure of medium sand slopes starts with cracks at crest of slope and takes the form of retrogressive toe sliding failure. With the increase of fine particles in the soil, the failure mode of the slopes changed to fluidized flow.

When the content of fine particle is less than 10%, the failure mode of granular debris flow is retrogressive toe sliding. The failure mode of granular debris flow is flow-slide failure when fine particle is more than 40%. And the failure mode of granular debris flow is the combine of retrogressive toe sliding and fluidized sliding when fine particle content is within the range of 20% and 30%.

A debris flow numerical model considering liquid-solid coupled method and nonsaturated soil cohesion has been established based on DEM of PFC^3D^. By comparing the flume model tests and numerical simulation, it was demonstrated that numerical model established by DEM is able to simulate the three-dimensional behavior of debris flow satisfactorily.

Based on the experimental test and numerical simulation, the failure modes of medium and fine sand slopes are represented as retrogressive failure and fluidized slide, respectively. The retrogressive failure of medium sand slope is tiered sliding. The fluidized slide of fine sand slope is whole body slip in a short time as viscous fluids. This characteristic of failure behavior of granular debris flows confirms well the experimental test and theoretical derivation.

## Figures and Tables

**Table 1:** Physical indices of the sand samples.

Sand type	Coefficient of uniformity (CU)	Coefficient of curvature (Cc)	Maximum dry density (*ρ*_max_)	Minimum dry density (*ρ*_min_)	Friction angle *ϕ* (°)
Medium sand	1.9	1.1	1.74 g/cm^3^	1.48 g/cm^3^	32.8
Fine sand	1.7	1.03	—	—	28.5

**Table 2:** Failure description of the sand slopes.

Test	Content of fine particles (%)	Failure mode	Description of failure
M	0	Retrogressive toe sliding	Crack develops at crest and starts to slide
C-10	10	Retrogressive toe sliding	Crack develops at crest and starts to slide
C-20	20	Combined	Crack develops at crest and slide takes near circular shape
C-30	30	Combined	Crack develops at crest and slide takes near circular shape
C-40	40	Fluidized flow	Surface settlement observed and sand slides rapidly
C-50	50	Fluidized flow	Surface settlement observed and sand slides rapidly
F	100	Fluidized flow	Surface settlement observed and fails to exhibit fluidized flow

**Table 3:** The coefficient of numerical model.

Particle	
Grain size/mm	2.0~5.0
Friction coefficient	0.5
*ρ* (kg/m^3^)	2650
Normal stiffness (N/m)	5.0*E* + 06
Wall	
Shear stiffness (N/m)	5.0*E* + 06
Normal stiffness (N/m)	1.00*E* + 07
Shear stiffness (N/m)	1.00*E* + 07
Friction coefficient	0.5
Fluid (water at 20°C)	
*ρ* (kg/m^3^)	1000
Viscous coefficient (Pa·s)	1.00*E* − 03
Flow net size (cm)	2.5 × 2.5 × 2.5
Flow solid parameters	
g (m/s^2^)	9.81
Time step DEM (s)	5.0*e* − 4
Time step CFD (s)	5.0*e* − 6
Viscous damping constant	
Normal	0.2
Tangential	0.2
